# PI3K/AKT Pathway and Brain Malformations

**DOI:** 10.15844/pedneurbriefs-29-7-3

**Published:** 2015-07

**Authors:** Gavin B. Rice, Nitin R. Wadhwani

**Affiliations:** 1 Department of Pathology and Laboratory Medicine, Ann & Robert H. Lurie Children's Hospital of Chicago, Chicago, IL; 2 Department of Pathology, Northwestern University Feinberg School of Medicine, Chicago, IL; 3 Furman University, Greenville, SC

**Keywords:** PI3K/AKT mutations, Cortical dysplasia, Megalencephaly

## Abstract

Investigators from Seattle Children's Research Institute, University of Washington, and collaborating institutions sought to evaluate 10 genes in the PI3K/AKT pathway as it relates epileptogenic brain malformations in patients with megalencephaly, hemimegalencephaly, and focal cortical dysplasia.

Investigators from Seattle Children's Research Institute, University of Washington, and collaborating institutions sought to evaluate 10 genes in the PI3K/AKT pathway as it relates epileptogenic brain malformations in patients with megalencephaly, hemimegalencephaly, and focal cortical dysplasia [1]. Malformations of cortical development (MCD) are common causes of intractable pediatric epilepsy and are usually diagnosed clinically, aided by imaging; however final classification is done by routine histopathology using the ILAE consensus classification [2].

They collected epileptic tissue specimens from 33 children with intractable epilepsy due to hemimegalencephaly (HMEG) and focal cortical dysplasia (FCD). Only peripheral blood was available from 1 child with bilateral HMEG. Of the 34 children, one had bilateral HMEG, and the rest had unilateral lesions (16 males, 18 females). 6 lesions were classified as HMEG; 27 as FCD (5 type I, 13 type IIa, 6 type IIb, and 3 type IIId). Of the 34 children, pathogenic mutations were found in 5 (including the bilateral HMEG patient): (1) PIK3CA mutation in the bilateral HMEG, (2) PIK3CA mutation in HMEG with linear epidermal nevus, (3) PIK3CA mutation in a localized FCD IIa, (4) AKT3 mutation in HMEG with cutaneous vascular malformations and unilateral ocular enlargement, and (5) germline PTEN mutation with HMEG. PI3K/AKT pathway activation as examined by western blot analysis showed an increase in phosphorylated AKT activity in the majority of HMEG and FCD tissue specimens. The downstream immunohistochemical marker for mTOR (phospho-S6) was positive in dysmorphic neurons. [1]

COMMENTARY. It makes intuitive sense that disruption of the mTOR (mammalian target of rapamycin) pathway may result in a continuum of changes ranging from cortical dysplasia to megalencephaly, however how many epileptogenic foci are truly caused by mutations? The mTOR pathway is influenced by numerous cell signals (insulin, growth factors, stress) and the principle pathway exerting influence is PI3K/AKT. As AKT phosphorylates mTOR directly, dysregulation of the mTOR pathway can result in abnormal cortical brain development as seen in patients with Tuberous Sclerosis. In addition, from the previous work of Riviere [3] we know that PI3K-AKT signaling plays an important role in vascular, limb and brain development; moreover, megalencephaly-associated mutations result in higher PI3K activity and PI3K-mTOR signaling. This was confirmed here.

In isolation, the individual findings are interesting, however when combined, the data suggests a shared pathogenesis for malformations of cortical development. At least for now, those institutions who receive such pathology specimens should utilize a biobank/biorepository. As the authors point out, more cases of HMEG will likely have alterations in the PI3K/AKT/mTOR pathway as compared to FCD. Most of the cases we see in clinical practice at our institution are FCD IIa. Only 1 case of FCD IIa in the current series was remarkable for a mutation in PIK3CA. Therefore, it is possible that other genes/growth factors are influencing the pathways that are outside the immediate realm of the PI3K/AKT/mTOR. Of note, no mutations were seen in the FCD I and FCD IIb cohort of specimens.

While our immediate practice will not be altered by the investigations of Jansen and colleagues, targeted testing of the PI3K/AKT pathway may disclose the etiology of epileptic lesions in patients with cortical dysplasia associated with other cutaneous lesions.
